# Neurotechnology: Current Developments and Ethical Issues

**DOI:** 10.3389/fnsys.2017.00093

**Published:** 2017-12-13

**Authors:** Oliver Müller, Stefan Rotter

**Affiliations:** BrainLinks-BrainTools, Albert Ludwigs University of Freiburg, Freiburg, Germany

**Keywords:** neurotechnology and brain-machine interface, ethics, self, personhood, deep brain stimulation

Neurotechnology is a fascinating and, at the same time, controversial field as one of its goals is to directly “wire up” human brains to machines. We should indeed expect to encounter such hybrid brain-machine systems more frequently in the future (see e.g., http://www.nature.com/nature/focus/brain/index.html). Neurotechnology is defined as the assembly of methods and instruments that enable a direct connection of technical components with the nervous system. These technical components are electrodes, computers, or intelligent prostheses. They are meant to either record signals from the brain and “translate” them into technical control commands, or to manipulate brain activity by applying electrical or optical stimuli. Closed-loop interactions of readout and stimulation systems (control circuits) are subject of current research as well. In the following, we would like to offer some insight into the current state of basic and applied research, and possible clinical applications resulting from it. We will also address some of the ethical issues that emerge in the context of neurotechnology and describe some ongoing interdisciplinary research on brain-machine interfaces.

Neurotechnological electrodes can be simply placed on the surface of the head in the form of electrode caps that pick up electrical fields generated by the active brain. This method of measurement is termed “non-invasive” as the electrodes do not penetrate the body. It is used, for instance, in patients suffering from amyotrophic lateral sclerosis (ALS), who are almost completely paralyzed during the advanced stages of the disease. These patients are sometimes only able to communicate using their eyelids or, alternatively, by voluntary changes of their electrical brain activity. In fact, these patients are still capable of controlling certain aspects of their measurable brain activity and, relying on suitable technical devices for decoding, can thus respond to yes/no questions. After some practice, they can operate a computerized “typewriter” and compose sentences. Their faculty of speech finds its way from the head directly to the computer.

Recording electrodes can yield more precise and more specific readouts if they are placed deep inside the brain, close to the nerve cells. Such methods are called “invasive” as the electrodes have to penetrate the brain tissue. They are considered for application if a complex device with many degrees of freedom, such as a prosthetic arm with an attached gripper or hand, is to be steered by brain activity. In such cases, the electrodes are often implanted into the motor cortex, an area of the brain that is normally responsible for controlling natural voluntary limb movements. In the USA, extended clinical tests of this spectacular technology have already been performed in a small number of patients. Beyond the development of neuroprostheses, this type of implantable neurotechnologies also opens new possibilities for the diagnosis of neurological diseases. For example, high-resolution grid electrodes have been developed, to be placed below the cranial bone, directly on the surface of the brain, in order to localize pathological excitation patterns in epilepsy.

Current research now seeks to optimize the long-term stability and biocompatibility of such brain implants to make them viable for everyday practical use beyond clinical trials. In order to further improve accuracy and reliability of such prostheses for patients, enhancements from modern robotics are increasingly considered by the engineers, and tools from machine learning are expected to make neuroprostheses adaptive and “intelligent.” Equipped with a certain degree of autonomy, they would be able to execute movements more aptly (e.g., not toppling any jars), or even detect which task is intended by the patient and execute it smoothly without detailed control. This new research is determined to come up with highly complex devices that will inevitably raise various serious ethical and even anthropological questions.

In addition to “readout” electrodes, there are also stimulating electrodes which are implanted into the brain to externally excite or inhibit specific nuclei, areas or fiber bundles using electric current or, more recently, with the aid of light. The electrodes for “deep brain stimulation” (DBS), for example, are inserted by a neurosurgeon with utmost precision into the respective regions deep in the brain. Through interference with these targets it is possible to suppress or ameliorate some symptoms of specific brain diseases. To some patients this can mean an enormous improvement of their clinical condition or subjective well-being. For example, DBS is regularly used in patients suffering from Parkinson's Disease (PD) if medication is ineffective. DBS can neither cure nor stop the progression of the neurodegenerative processes, but it can significantly alleviate typical severe symptoms such as tremor or rigor, this way significantly improving the patient's condition and quality of life. DBS is meanwhile also considered for application to other neurological diseases, such as epilepsy or Tourette syndrome, and even for the treatment of certain psychiatric conditions, such as major depressive disorder (MDD) (Holtzheimer and Mayberg, 2011; Fitzgerald and Segrave, 2015). A somewhat futuristic variant of stimulation uses innovative methods from optogenetics: nerve cells are made photosensitive by inserting artificial light receptors into their membranes, not unlike receptor cells in the eye's retina. Illumination with light of adequately chosen wavelengths then leads to either excitation or inhibition in these cells, which can be easily controlled externally by simply turning the light source on and off (Pashaie et al., 2014; Warden et al., 2014). These new techniques of stimulation with light—also suggested for innovative hearing aids, or targeted inhibitory “counter steering” in case of an epileptic seizure—face some severe disadvantages, however. Ultimately, it is a genetically engineered manipulation that makes “normal” nerve cells expressing light receptors. The required modifications to the cells' genetic information are efficiently carried out by manipulated viruses—with all the risks and side effects that may come with such an intervention. Much additional research and methodical refinement will be required until this procedure, which has been successfully tested in animals for many years now, can also be safely applied in humans, with calculable side effects.

A whole new spectrum of possibilities arises when neurotechnologies for recording and stimulation are employed simultaneously. The great potential of such a combination of techniques comes from the observation that an electric stimulus may have entirely different effects, depending on the activity state of the brain tissue at stimulation time (Rosin et al., 2011; Berényi et al., 2012; Cagnan et al., 2017). Imagine a child sitting on a swing: It will learn without effort how by moving one's legs in the right moment (“stimulation”) one can intensify, or attenuate the undulation of the swing (“activity”). In order to improve efficiency and reduce inevitable side effects of the interference, the idea is to make electrical stimulation dependent on the actual brain activity. The latter is recorded online, thus informing the controller and allowing it to apply the stimulation in the right moment. This closes the loop: Stimulation modifies activity, activity influences stimulation. Since control must be exerted promptly and precisely, this task is typically assigned to a dedicated signal processor. With this, implants will not only include electrodes for measurement and stimulation, but also the computing device that is required for control, or at least the interface to an external computer. The joint operation of metal and silicon in the brain, possibly enhanced by implantable wireless technology, will enable completely new applications in the future that go far beyond current possibilities.

Neurotechnology-based interference with brain activity can be very effective, allowing for successful treatment of brain disorders. This approach complements traditional (mostly pharmaceutical) treatment methods, and it often leads to a substantial improvement in quality of life. However, one has to understand that these interventions change the brain and its functions—either as a desired result of therapy, or as an unwanted side effect. In extreme cases, interventions in the brain can transiently or irreversibly alter a patient's personality and character. This is of course intended in the treatment of certain affective disorders. However, changes to personality can also be an unintended side effect of brain intervention, as occasionally reported in PD patients receiving DBS. How far should we go when cognitive and emotional alterations of a person could result from an intervention? Which kinds of risks are acceptable? Does our “self” change into another one by these interventions? Are we the same person we were before the operation and before the stimulation? Does our notion of legal “responsibility” change if intelligent neuroprostheses autonomously interpret or even change our brain activity? (see for an overview about the ethical questions Roskies, 2002; Glannon, 2006; Illes and Sahakian, 2011; Clausen and Levy, 2015).

Neurotechnology raises ethical questions that are associated with what we call our “self” or “soul,” complex philosophical concepts with many presuppositions (Vogeley and Gallagher, 2011). The ethical debate usually draws on the concept of personhood as a “modern” notion that includes core aspects that we typically ascribe to our self or soul (Merkel et al., 2007). These include self-consciousness, responsibility, planning of the individual future, and similar dimensions. In our deliberations, we should first make ourselves aware of the notions of “person” and “personal identity” as fundamental concepts of ethics. Integrity and dignity of a person are the most relevant criteria for the ethical evaluation of technological interventions. The concept of personhood always has normative implications, because we not only describe certain attributes and capabilities of a person, but we want to have them recognized, acknowledged and guaranteed. For example, the principle of “informed consent,” which is so important in clinical practice, refers to the notion of personhood (cf. Beauchamp and Childress, 2008). Patients must consciously authorize a neurotechnological intervention before it is conducted. Along similar lines, the concept of a person can provide an ethical benchmark, assuming that we do not want to impair personal capabilities such as autonomy and responsibility by interventions in the brain. Neurotechnological interventions are ethically not acceptable if remaining a person is at risk. The current practice of neurotechnological interventions is, explicitly and implicitly, orientated toward the concept of personhood.

Yet, the situation is more complicated—as it is so often the case in ethical evaluations (Schermer, 2011). Although a patient typically does remain a person after an intervention in the strict philosophical sense of the term, he or she could be left with an altered personality, with unfamiliar character traits and new or previously subliminal behavioral patterns. Upon the use of DBS in PD patients, an altered personality can be diagnosed in many cases. Some of them are subtle, but they may also be quite severe. We have seen the onset of a depressive disorder that had not existed prior to the intervention. There are cases of disproportionate euphoria occurring in patients, who before DBS onset had been known for their “rational” behavior, but are now inclined to risky financial decisions, for example. What we observe here is not so much an impairment of personhood, but alterations of personality and character traits.

Is the patient's personal identity threatened in these cases? The concept of personal identity refers to the question of to which degree and under which circumstances a person remains the same over time, above and beyond physical identity. Answering this question requires that we develop concepts and provide criteria which allow us to establish the “sameness” of a person over time, a complicated problem that is ethically relevant, however. Not only the interaction with other humans, but also the appreciation of moral capabilities—such as the ability to make a promise and keep it—are firmly rooted in the assumption that we and the others remain “the same” beyond any doubt. In the international debate on this (see Baylis, 2013), there is the tacit assumption that, even in the face of distinct and visible personality changes, personal identity is not compromised. Drawing on concepts of narrative identity we can assume the “sameness” of a person, because human beings experience themselves as being the same though the narrative of their life history. Even the sizeable gaps implicated by illness or a debilitating therapy such as DBS are perceived as an integral part of one's own history, of one's identity. In his book *Deep in the Brain*, the sociologist Helmut Dubiel elaborated on his personal experience with Parkinson's Disease and DBS (Dubiel, 2009). His struggle to understand the technology in his head, and how it affects his daily life, can be considered an example of how neurotechnology can be integrated in someone's life, his experiential horizon, his self-concept and self-image. Even in such a severe case, personal identity is not put in question at all. Despite all personal distortions and weird experiences, Dubiel remains the same.

Yet, there are also examples where this constancy can no longer be assumed. Medical ethicist Walter Glannon describes the story of a patient who, after having undergone DBS, entered a state of euphoria such that his family could no longer recognize him as the one they knew before (Glannon, 2009a). The patient himself, however, felt very happy in his condition; not only were the negative symptoms of Parkinson's Disease suppressed to a large extent, but also did he just feel “happier” as a result of his stimulation-induced mania. When a decision had to be made as to whether he should be admitted to a mental institution as he could no longer live on his own, several dilemmas became apparent: In which “state” should a person be asked for his informed consent on a treatment? Should the patient be consulted before or after the stimulation in cases like the one reported above? Which of the patient's “states” qualifies him or her as “self-responsible”? But we must also take into account the family environment and the health care system: How much “alienation” must relatives accept? Should society cover the costs for hospitalization?

The situation described by Walter Glannon is certainly rather extreme, and it fortunately occurs only very rarely. From an ethical perspective, it will be important in the future to accurately grasp and understand subtle and less subtle alterations caused by neurotechnological interventions in the brain so that generally accepted ethical standards can be developed. This requires the integration of several perspectives. The psychological qualitative and quantitative assessment of personality alterations is one of them. However, we also need new descriptive categories to grasp the specifics of a technology that has the potential to affect the daily life of many. Patients must learn to frequently “switch” between different states of their own personality, as the stimulator can be turned on and off at the push of a button. Neurotechnological intervention concerns our self, which is not simply the consequence of the activity of our brain, but it arises from an interaction between our body and the world, including our social and cultural environment. This is why the ethical evaluation of neurotechnologies also needs to introduce the critique of what has been termed “neuroreductionism” or “neuroessentialism” (see e.g., Fuchs, 2006; Glannon, 2009b). The self, the I, the person is more than the brain and its functions. An anthropological critique of reductionist positions is also ethically relevant, with regard to a multi-dimensional and anthropologically grounded concept of personhood. The large body of neurobiological knowledge about pathological processes in the brain considered, we are after all still dealing with persons that suffer from a disease, and not just with a dysfunctional organ. This also bears great significance for clinical practice. If the brain is regarded as an isolated organ in the treatment of a disease, and our self is deemed just an “appendage” of brain activity, it is easy to forget that different forms of therapy need to be integrated here. Interventions enabled by neurotechnology are ethically justified, if and only if the successful treatment of very serious brain diseases follows from them. In the future, however, ethical assessments will also have to bear in mind the “mechanization” of the self through neurotechnological interventions, and the consequences this has for the everyday life of human beings. In this regard, we will enter uncharted territory which must be thoroughly surveyed.

The fascinating aspect of neurotechnology is that, in a certain sense, human beings and machines are “fused” together to a degree unheard of before. We could not possibly provide a definition of “human being” without a comprehensive notion of technology—humans have always been “artificial” beings as they have always relied on technology, emphasized by the term “homo faber.” Even though our traditional “replacement prosthetics” and assorted bodily enhancements provide us with a rich repository of experiences with forms of self-mechanization, the direct implantation of silicon into the brain constitutes an entirely new form of mechanization' of the self. This not only concerns the alterations of personality discussed earlier—the new union of man and machine is bound to confront us with entirely new challenges as well. Basic research is performed on robotic arms that can “autonomously” interpret and execute the patient's motor intentions. To this end, the research toward decoding people's intentions from brain activity will be intensified. The goal is that a neuroprosthesis “knows” which of the elevator buttons the patient in the wheelchair wants to press. As we can retrieve ever more detailed and voluminous information about what is going on “inside” a patient, the issues of data integrity, data security, and privacy are gaining very high relevance for neurotechnology as well. Naturally, the read-out of brain activity and the corresponding data processing help the patient and alleviate the consequences of a disease or disability, thus restoring his or her quality of life to some degree. However, these data also become more “sensitive” the more precisely one is able to interpret the patients' intentions and internal states. The impact of an unintended manipulation of such brain data, or of the control policy applied to them, could be potentially harmful to the patient or his/her environment. And if office computers, mobile telephones, and industrial facilities can be “hacked” and taken over by computer viruses or Trojans—why should this not be possible for a neuroprosthetic device as well? It will take a few more years until the new interfaces have been thoroughly tested for reliability and viability. But we can already now envision that our concept of “responsibility” will undergo dramatic changes, if the users' intentions are transferred to a machine. Computer-based translation and technical implementation transform the user's identity: He is now man and machine at the same time. The next few years will see a need for elaborating ethical and legal frameworks that stimulate and regulate responsibility on both the human and the artificial side (including the machine's manufacturer) such that a man-machine complex can be safely integrated into daily life (Kellmeyer et al., 2016).

Current developments in stimulation technology based on optogenetics raise ethical concerns, not only regarding the acceptability of interventions in the brain and their consequences, but also in view of the necessary genetic modifications of the organism. In order to make stimulation through light possible one must reprogram cells with the help of manipulated viruses. This calls for an assessment of more than just risks and benefits. Such interventions challenge the very ethical self-understanding of the doctors who are to perform the targeted genetic modification of their patients before the symptoms of their illnesses can be treated by optic electrodes. What must be evaluated here is whether the advantages of optical stimulation vs. electrical stimulation—which also imposes irreversible damage to neural tissue—can be compensated by the modification of genetic information, particularly considering that these techniques entail long-term risks that are difficult to assess.

We are moving toward a future where the artificial and the organic, the “human” and the “mechanical” will interact more directly than ever, where invasive technical interventions will reach the brain. The future will show whether we are to become cyborgs at some point, and what we will see when looking back on present-time neurotechnology. But the debate on whether and how we should make our brains ready to be “plugged” to technical devices must begin today. We must discuss which are the risks we are willing to take—and whether there are paths in this uncharted territory that we may not wish to enter.

## Author contributions

OM was responsible for the ethical parts of this interdisciplinary article. SR for the neuroscientific and technological aspects. But we collaborated intensively in the development of the ideas.

### Conflict of interest statement

The authors declare that the research was conducted in the absence of any commercial or financial relationships that could be construed as a potential conflict of interest. The handling Editor declared a shared affiliation, though no other collaboration, with the authors [OM and SR].
